# Neurodegenerative Evidence in Mice Brains with Cecal Ligation and Puncture-Induced Sepsis: Preventive Effect of the Free Radical Scavenger Edaravone

**DOI:** 10.1371/journal.pone.0051539

**Published:** 2012-12-07

**Authors:** Hiroki Yokoo, Seiichi Chiba, Kengo Tomita, Michinori Takashina, Hiroshi Sagara, Saburo Yagisita, Yasuo Takano, Yuichi Hattori

**Affiliations:** 1 Department of Molecular and Medical Pharmacology, Graduate School of Medicine and Pharmaceutical Sciences, University of Toyama, Toyama, Japan; 2 Department of Internal Medicine, Faculty of Medicine, Oita University, Yufu, Japan; 3 Medical Proteomics Laboratory, Institute of Medical Science, University of Tokyo, Tokyo, Japan; 4 Section of Pathology, Kanagawa Rehabilitation Center, Atsugi, Japan; 5 Kanagawa Cancer Research Institute, Yokohama, Japan; University of São Paulo, Brazil

## Abstract

Sepsis is a major clinical challenge and septic encephalopathy is its nasty complication. The pathogenesis and underlying mechanisms of septic encephalopathy are not well understood. This study sought to fully characterize sepsis-associated biochemical and histopathological changes in brains of mice after cecal ligation and puncture, regarded as a highly clinically relevant animal model of polymicrobial sepsis. Real-time PCR analysis showed that gene expression levels of proinflammatory cytokines, including tumor necrosis factor-α and interleukin-1β, were significantly up-regulated in brain tissues from septic mice, but to a much lesser extent when compared with those in peripheral tissues such as lungs. Blood-brain barrier (BBB) permeability was significantly increased in septic mice, as determined by the measurement of sodium fluorescein and Evans blue content. Sepsis resulted in increases in NADPH oxidase activity and expression of p47*^phox^* and p67*^phox^* and up-regulation of inducible nitric oxide (NO) synthase in brains, indicating that superoxide, produced by NADPH oxidase, reacts with NO to form peroxynitrite, that maybe lead to the loss of BBB integrity. Light and electron microscopic examination of septic mouse brain showed serious neuronal degeneration, as indicated by hyperchromatic, shrunken, pyknotic, and electron-dense neurons. These histopathogical changes were prevented by treatment with the free radical scavenger edaravone. Together, these results suggest that sepsis can lead to rapid neurodegenerative changes in brains via free radical species production and possibly subsequent injury to the BBB. We may also provide a potentially useful therapeutic tool for treating septic encephalopathy.

## Introduction

Sepsis is a systemic inflammatory response syndrome based on the presence of infection and is a major clinical challenge in intensive care medicine. Despite recent progress in antibiotic and critical care therapy, sepsis presents a very high mortality rate [1], [2]. Sepsis is a complex syndrome with its wide spectrum of severity. Septic shock is a subset of severe sepsis defined as hypotension that is poorly responsive to fluid resuscitation. Furthermore, multiple organ dysfunction is a continuum, with incremental degrees of physiological derangements in individual organs, and has a significant clinical impact [3].

In sepsis, the brain may be affected by many systemic disturbances, such as hypotension, hypoxemia, hyper- or hypoglycemia, and organ dysfunction. Thus, brain dysfunction is often one of the first clinical symptoms in sepsis. Septic encephalopathy is a symmetric diffuse brain dysfunction and remains a baffling complication of the sepsis syndrome with limited therapeutic options. The neurological manifestations can range from mild confusion and agitation, often evident in elderly patients with serious infection, to frank delirium, stupor, or coma in life-threatening cases [4], [5]. Septic encephalopathy is the most common form of encephalopathy among patients in intensive care units. The incidence of septic encephalopathy varies between 9 and 71% of septic patients, probably depending on the different definitions of sepsis and encephalopathy used [6]. The onset of encephalopathy often precedes failure of other organs such as lung, liver, and kidney, and it is associated with significantly increased mortality [7].

The pathophysiology of septic encephalopathy is not well understood. Various mechanisms proposed for the pathogenesis of septic encephalopathy involve cerebral edema and inflammation, inflammatory cells and their mediators, blood-brain barrier (BBB) disruption, reduced cerebral blood flow, microvascular disorders such as microinfarction and microthrombus, impairment of astrocytes and neurons, neurotransmitter derangement, apoptosis, oxidative stress, and calcium deregulation [5], [8], [9]. Accordingly, since septic encephalopathy may be the consequence of multifactorial pathogenesis, there are significant limitations on mechanism-based therapies to prevent or cure this disorder.

**Figure 1:**
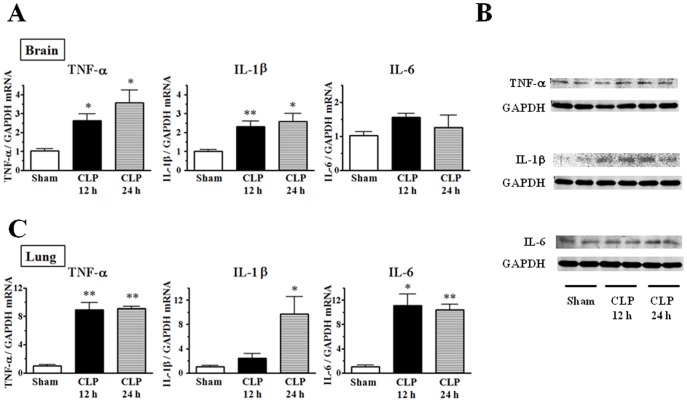
Sepsis-induced up-regulation of proinflammatory cytokine expression in brain and lung tissues. Tissues were harvested 12 and 24 h after CLP. In (A) and (C), the mRNA levels for TNF-α, IL-1β, and IL-6 were quantified by real-time PCR. The data are expressed as a fold increase above control (sham operation) normalized GAPDH. Mean of data from 3–5 animals in each group is presented, with SEM by vertical lines. *p<0.05, **p<0.01 versus sham-operated control. (B) Western blots of TNF-α, IL-1β, and IL-6. GAPDH served as loading control. Representative images from two separate experiments are shown.

Cecal ligation and puncture (CLP) is a clinically relevant model of sepsis and lethal systemic inflammation [10]–[12]. It is similar to the clinical situation of bowel perforation, inducing peritonitis due to mixed intestinal flora. The animals subjected to CLP generally become severely hypotensive without an apparent hyperdynamic phase [13] and show an inflammatory cytokine profile similar to that in human sepsis [14]. In this study, we evaluated sepsis-associated histopathogical changes in the brain by using a CLP mouse model. Furthermore, we aimed at identifying a possible therapeutic target for preventing the histopathogical changes found in septic brains.

**Figure 2:**
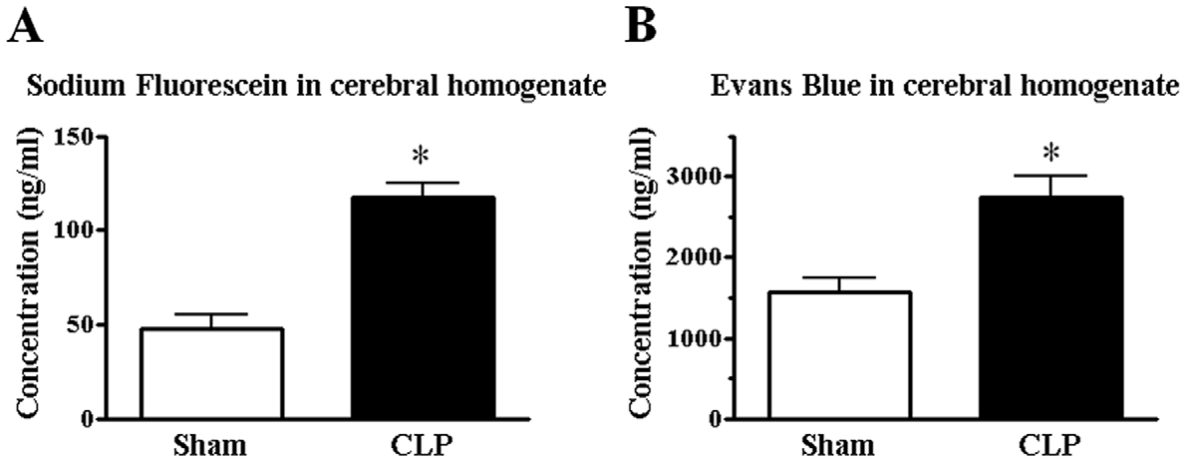
Sepsis-induced increase in extravasation of sodium fluorescein (A) and Evans blue (B) in brains. Tissues were harvested 24 h after surgery. Mean of data from 4–6 animals in each group is presented, with SEM by vertical lines. *p<0.05 versus sham-operated control.

## Materials and Methods

### Model of Sepsis

Mice care, experimental protocols, and monitoring for suffering were treated according to the National Institute of Health Guidelines on the use of laboratory animal and with approval of the Animal Care and Use Committee of the University of Toyama. Male BALB/c mice, 8 to 12 weeks of age, were maintained under constant temperature (22±2°C), humidified (55±10%), and lighting conditions (lights on 7 AM to 7 PM). Mice were allowed *ad libitum* access to food and water throughout quarantine. The surgical procedure to generate CLP-induced sepsis was performed according to the method described in our previous reports with minor modification [15], [16]. Mice were anesthetized with 3 to 4% sevoflurane, and a middle abdominal incision was made. The cecum was mobilized, ligated at 5 mm from its top, and then perforated in two locations with a 21-gauge needle, allowing exposure of feces. The bowel was repositioned, and the abdomen was closed with sterile suture. Sham-operated control animals were subjected to the same surgical laparotomy, but the cecum was neither ligated nor punctured. All CLP mice were lethargic, showed lack of interest in their environment, displayed piloerection, and had crusty exudates around their eyes, as contrasted with sham-operated mice that were healthy, moving freely and eating [17]. At each experimental time point from the procedure, mice were sacrificed by perfusing solution under 3 to 4% sevoflurane anesthesia. Mice with extreme mobility, which exceeded the level of the predetermined score as to the welfare, were euthanized under sevoflurane anesthesia even before the scheduled time point.

**Figure 3:**
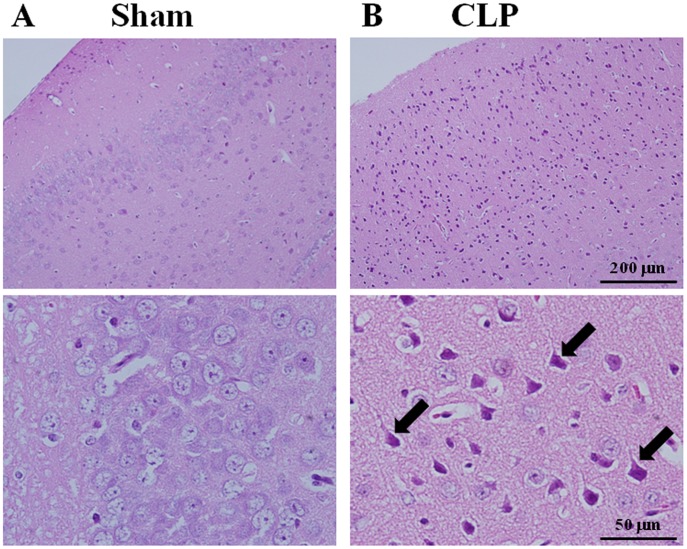
Morphological damage of neurons in cerebral cortices from septic mice. Images from hematoxylin and eosin-stained sections represent cerebral cortices from sham-operated control (A) and from CLP septic mice (B). Tissues were harvested 24 h after surgery. Bottom shows high-magnification images. Arrows indicate densely-stained cells. Shown are representative micrographs from three independent experiments in which the same results were obtained.

### Drug Administration

Some mice were injected edaravone (Mitsubishi Tanabe Pharma, Osaka, Japan) intraperitoneally at a dose of 10 mg/kg twice a day. This dose was chosen accordingly to previous literatures showing evaluation of edaravone effects on cerebral ischemic stroke and cardiac hypertrophy in rodents [18]–[20]. Edaravone, 150 mg, was dissolved in 2.5 ml of 1 N NaOH, the pH was adjusted to 7.0 with 1 N HCl, and the dosage of 3 mg/ml was prepared by adding saline [21]. Edaravone treatment was begun from 4 days before the surgical procedure and was continued up to the day when the animals were killed. The placebo group was administered an equal volume of saline. We confirmed that vehicle treatment was without effect in control experiments.

**Figure 4:**
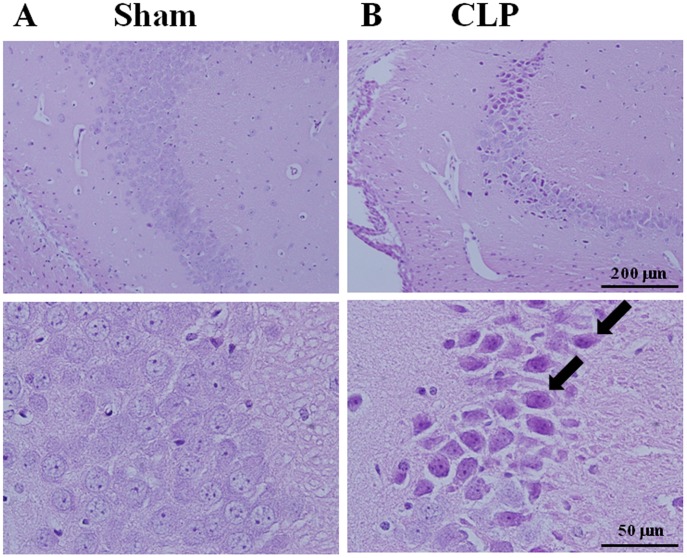
Morphological damage of neurons in hippocampal areas from septic mice. Images from hematoxylin and eosin-stained sections represent hippocampal areas from sham-operated control (A) and from CLP septic mice (B). Tissues were harvested 24 h after surgery. Bottom shows high-magnification images. Arrows indicate densely-stained cells. Representative micrographs from two independent experiments are shown.

### RNA Extraction and Quantitative Real-time PCR

Total RNA was isolated from brain and lung tissues with the use of TRI Reagent® (Sigma-Aldrich, St. Louis, MO) according to the manufacturer’s instructions. After incubation with DNase I (Thermo Fisher Scientific, Rockford, IL), reverse transcription was performed by using PrimeScript® RT reagent Kit (Takara Bio, Otsu, Japan) with 0.5 µg RNA in a 10 µl reaction mixture. An Mx3000P Real Time QPCR system (Stratagene, La Jolla, CA) was used for real-time PCR. PCRs were performed by using a SYBR® *Premix Ex Taq*™ II (Takara Bio) with 2 µl cDNA and 0.8 µl each primer (10 µM) in a total volume of 20 µl. The sequences of specific primer pairs for target genes (tumor necrosis factor-α [TNF-α], interleukin-1β [IL-1β], interleukin-6 [IL-6], p47*^phox^*, and p67*^phox^*) are available upon request from a website of Takara Bio (http://www.takara-bio.co.jp/). The PCR program consisted of 95°C for 30 s for initial denaturation of DNA, followed by 40 cycles of shuttle PCR reaction, 95°C for 5 s and 60°C for 34 s. Each sample was run in duplicates. Glyceraldehyde-3-phosphate dehydrogenase (GAPDH) served as a housekeeping gene to normalize the expression of target genes. Relative expression was calculated according to the 2^−ΔΔCt^ method as previously described [22].

**Figure 5:**
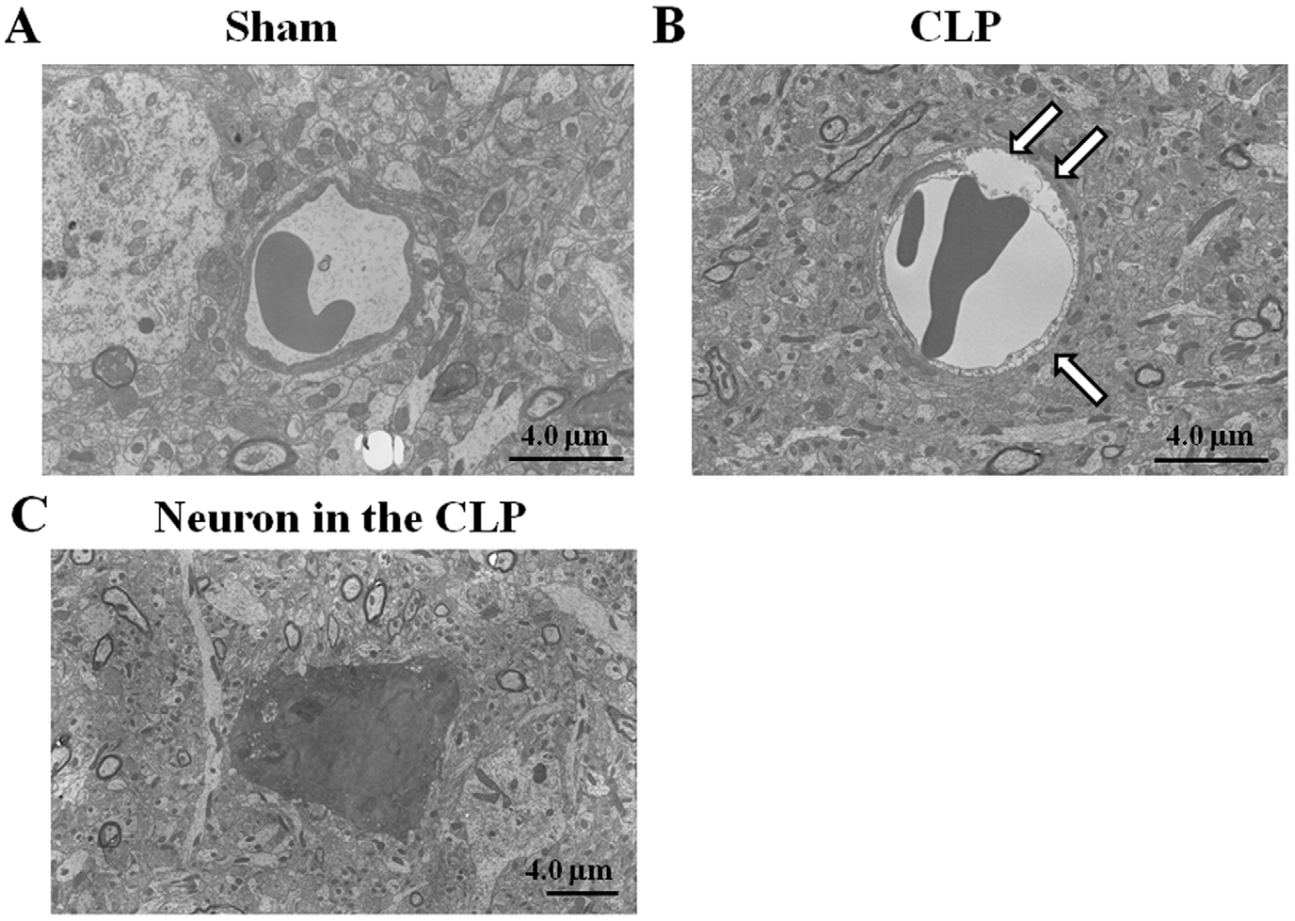
Ultrastructure of capillary and neuronal changes in cerebral cortices from septic mice. Images show normal-appearing capillary in the sham-operated group (A), markedly swelling capillary with partial disruption of the cytoplasmic membranes of endothelial cells (arrows) in the CLP-septic group (B), and electron-dense neuron in the CLP septic group (C). Tissues were harvested 24 h after surgery. Representative images from two separate experiments are shown.

### BBB Permeability

Assessment of BBB permeability was performed using the two tracers sodium fluorescein (molecular weight of 376 Da) and Evans blue (molecular weight of 961 Da). Both tracers can be used since sodium fluorescein represents the leakage of low molecular weight molecules into the brain, whereas Evans blue represents the leakage of high molecular weight molecules into the brain [23]. Evans blue has a high affinity for plasma protein albumin in blood circulation, which gives rise to a high-molecular complex (Evans blue-albumin, 68,500 Da) with limited penetration of the BBB under normal conditions. Mice were injected into tail vein with both tracers (20 mg/kg). Thirty min later, the animals were anesthetized with sevoflurane and were perfused with heparinized saline (1 U/ml) via the left ventricle. Then, the animals were killed by decapitation and the brains were quickly removed. The semi-cortices were dissected, weighed, and mechanically homogenized in 7.5% (W/V) trichloroacetic acid, and the homogenate of each sample was adjusted to 1 g/3 ml. Two aliquots (300 µl) were taken for measurement. One aliquot was neutralized with 50 µl 5 N NaOH and measured by fluorimetry (excitation 485 nm, emission 538 nm) for sodium fluorescein determination. The other was centrifuged for 10 min at 10,000×*g* at 4°C, and the supernatant was measured by absorbance spectroscopy at a wavelength of 620 nm for Evans blue determination. All measurements were within the range of detection established by the standard curve.

**Figure 6:**
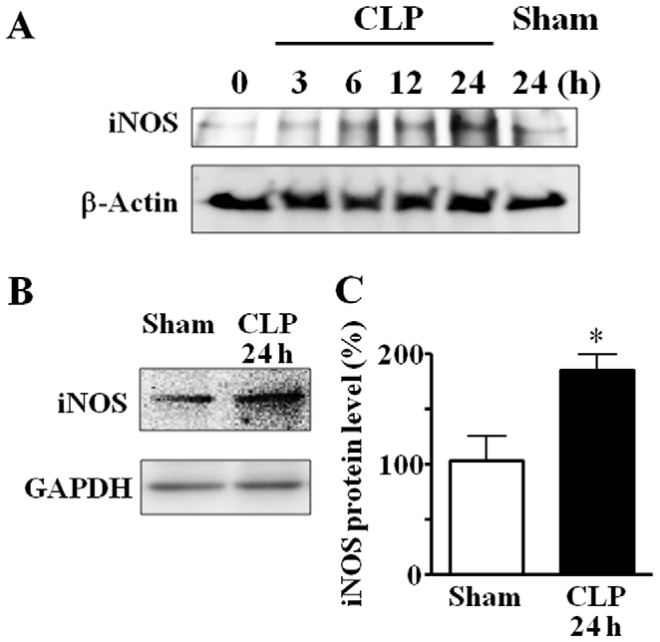
Sepsis-induced increase in iNOS protein expression in brain tissues. (A) Time course of changes in cerebral expression of iNOS protein after CLP. β-Actin served as a loading control. The result is representative of two independent experiments. (B) Comparison of iNOS protein expression between sham-operated and CLP septic groups at 24 h after surgery. The summarizing data are presented as iNOS/GAPDH expressed relative to the unoperated control. Mean of data from three animals in each group is presented, with SEM by vertical lines. *p<0.05.

### Histological Examination

The brains were fixed by immersion in 10% buffered formaldehyde overnight, embedded in paraffin, and cut into 5-µm-thick coronal sections. After deparaffinization, brain sections were stained with hematoxylin and eosin for routine histological evaluation. Cell shape and number were quantified using the measurement and analysis software VH-HIA5 (Keyence, Osaka, Japan).

**Figure 7:**
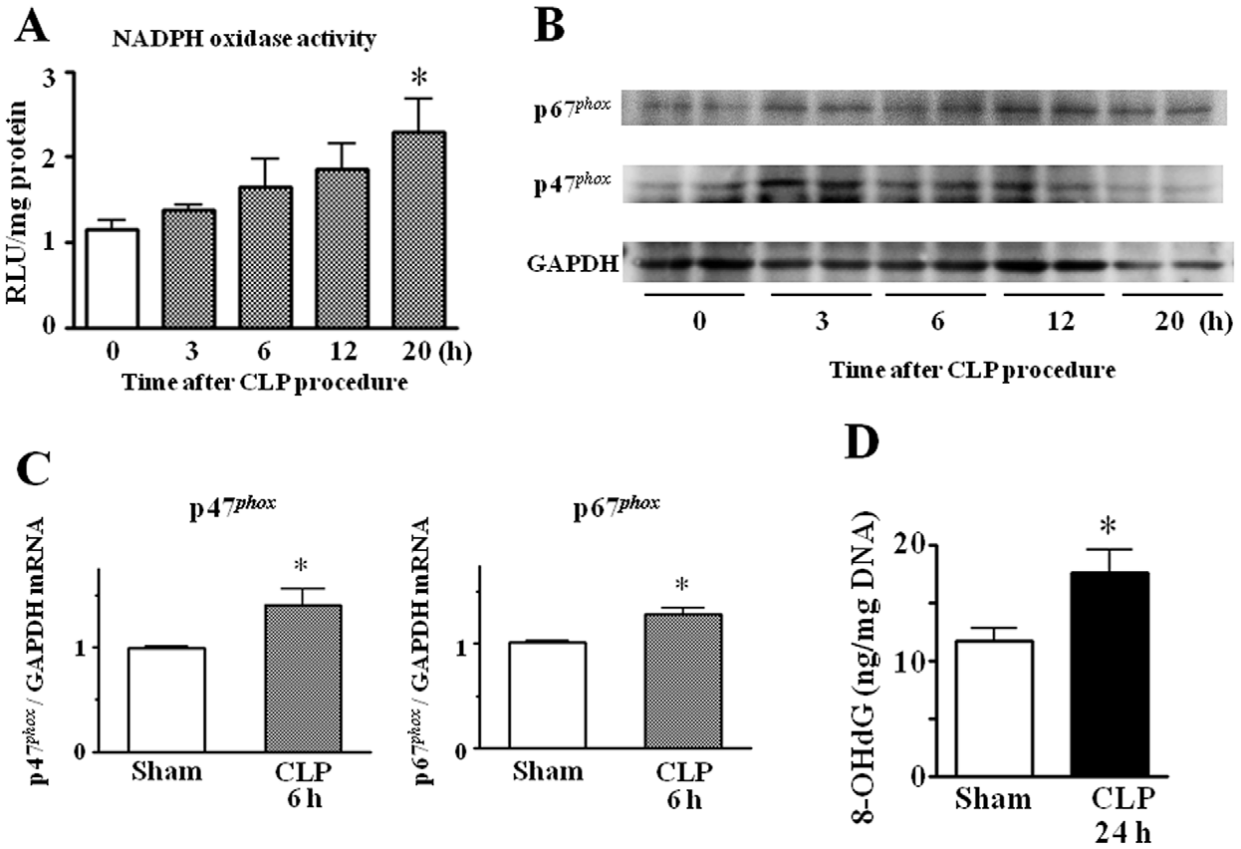
Sepsis-induced changes in NADPH oxidase activity, NADPH oxidase components p47*^phox^* and p67*^phox^* expression, and oxidative stress parameter in brain tissues. (A) Time course of changes in NADPH oxidase activity. Mean of data from six animals in each group is presented, with SEM by vertical lines. *p<0.05 versus control. (B) Western immunoblotting showing up-regulation of p47*^phox^* and p67*^phox^* protein expression after CLP. The result is representative of two independent experiments. (C) The mRNA levels for p47*^phox^* and p67*^phox^* were quantified by real-time PCR. The data are expressed as a fold increase above control (sham operation) normalized GAPDH. Mean of data from six animals in each group is presented, with SEM by vertical lines. *p<0.05 versus sham-operated control. (D) Levels of 8-OHdG. Mean of data from ten animals in each group is presented, with SEM by vertical lines. *p<0.05 versus control.

### Electron Microscopic Analysis

The brains were fixed by immersing in 0.1 M sodium phosphate buffer (pH 7.4) containing 4% formaldehyde, 2.5% glutaraldehyde for 2 h at room temperature, which were postfixed with 2% osmium tetroxide in the phosphate buffer. Samples were dehydrated through a graded series of ethanol and embedded in Epon 812 resin mixture (TAAB, Berks, U.K.). Ultrathin sections (∼70 nm) were cut by use of a Leica EM UC6 ultramicrotome (Leica, Wezlar, Germany), doubly stained with uranyl acetate and lead citrate, and examined with an H7500 electron microscope (Hitachi, Tokyo, Japan).

**Figure 8:**
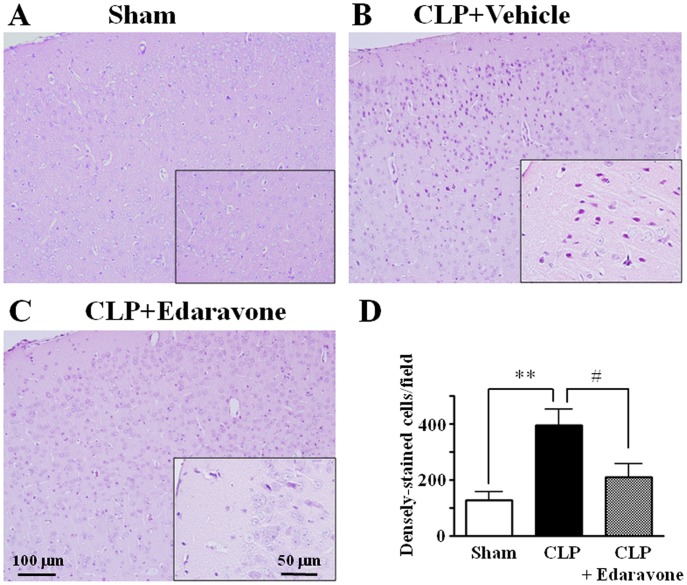
Effect of edaravone treatment on neuronal degeneration in cerebral cortex after sepsis. In the vehicle-treated CLP septic group, there was serious neuronal degeneration in cerebral cortex (B) compared with sham control (A). In the edaravone-treated CLP septic group, normal-appearing neuronal findings were obtained in cerebral cortex (C). Tissues were harvested 24 h after surgery. Magnified view is shown in each inset. (D) Estimation of densely-stained cells in cerebral cortex. Counts of densely-stained cells were made in the sections at a final magnification of ×100. Mean of data from eight animals in each group is presented, with SEM by vertical lines. **p<0.01 versus control. #*p*<0.05 versus CLP treated with vehicle.

### Western Blot Analysis

Mouse brain lysates were prepared by homogenization in ice-cold phosphate-buffered saline containing a protease inhibitor cocktail (Sigma-Aldrich). Protein concentrations were measured with a Bio-Rad protein assay kit (Bio-Rad Laboratories, Hercules, CA), based on the method of Bradford. Immunoblotting was performed as described in our previous reports [16], [24]. Samples (10 µg of protein) were run on 7.5, 12 or 14% SDS-polyacrylamide gel and electrotransferred to polyvinylidine difluoride filter membrane. Membranes were probed with anti-inducible nitric oxide synthase (iNOS) (OxisResearch, Portland, OR), anti-p47 phox (Millipore, Billerica, MA), anti-p67 phox (Millipore), anti-β-actin (GeneTex, San Antonio, TX), anti-TNF-α (Santa Cruz Biotechnology, Santa Cruz, CA), anti-IL-1β (Santa Cruz Biotechnology), anti-IL-6 (Santa Cruz Biotechnology), or anti-GAPDH (Santa Cruz Biotechnology). Primary antibody detection was performed with horseradish peroxidase-conjugated or IRDye®-labeled secondary antibodies. Binding of the antibody was detected by an ECL Plus chemiluminescent system (GE Healthcare, Tokyo, Japan) and levels of protein expression were quantitated by a luminoimage LAS-3000 analyzer (Fuji Film, Tokyo, Japan). Fluorescent of IRDye was analyzed by Odyssey® CLx Infrared Imaging System (LI-COR Bioscience, Lincoln, NE).

**Figure 9:**
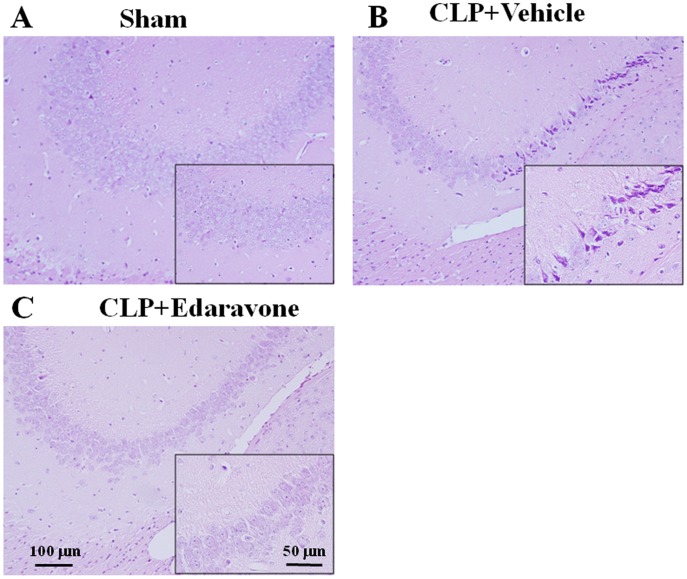
Effect of edaravone treatment on neuronal degeneration in hippocampus after sepsis. In the vehicle-treated CLP septic group, there was serious neuronal degeneration in hippocampus (B) compared with sham control (A). In the edaravone-treated CLP septic group, normal-appearing neuronal findings were obtained in hippocampus (C). Tissues were harvested 24 h after surgery. Magnified view is shown in each inset. Shown are representative micrographs from two independent experiments in which the same results were obtained.

### Measurement of NADPH Oxidase Activity

Brain tissues were homogenized on ice in a modified Krebs-HEPES buffer (119 mM NaCl, 20 mM HEPES, 4.6 mM KCl, 1.2 mM CaCl_2_, 1.0 mM MgSO_4_, 0.4 mM Na_2_PO_4_, 5 mM NaHCO_3_, and 5.5 mM glucose, pH 7.4) containing a protease inhibitor cocktail. Homogenates were centrifuged at 1,000×*g* at 4°C for 5 min. Supernatant of 100 µl was used for measurements of NADPH oxidase activity with the method of lucigenin chemiluminescence in the presence of 10 µM lucigenin and 100 µM substrate NADPH. No enzymatic activity was detectable in the absence of NADPH. Chemiluminescence was measured for 10 s by using a sensitive luminometer (TD-20/20; Turner Designs, Sunnyvale, CA). The cycle was repeated twice, and the three values were averaged. The results were reported at relative light units per minute.

### Measurement of 8-OHdG Levels

8-Hydroxydeoxyguanosine (8-OHdG), a modified DNA base product generated by free radicals, is considered to be a good biomarker of oxidative DNA damage. DNA was extracted from brains using a DNA Extractor TIS Kit (Wako, Osaka, Japan), which contain NaI as a chaotropic agent to cause less oxidation of DNA. Cerebral 8-OHdG levels were measured using an enzyme-linked immunosorbent assay kit (Highly Sensitive 8-OHdG Check; Japan Institute for the Control of Aging, Fukuroi, Japan) after preparation with an exclusive kit (8-OHdG Assay Preparation Reagent Set; Wako). The DNA was mixed with 8-OHdG monoclonal antibody, incubated at 4°C overnight and rinsed with 250 µl of washing solution. Enzyme-labeled secondary antibody (100 µl) was incubated with the DNA at room temperature for 60 min and rinsed with 250 µl of washing solution, followed by a 15-min incubation at room temperature with 100 µl of reconstitution enzyme substrate. The reaction was stopped by adding 100 µl of reaction termination solution and then absorbance was measured at 450 nm.

### Statistical Analysis

When appropriate, data are presented as the mean ± SEM. Statistical analysis was performed using Prism software (ver. 4; GraphPad Software Inc., San Diego, CA). Comparisons of more than three data were determined by one-way ANOVA followed by the Tukey's multiple comparison test. Student's *t* test was used when two means of group were compared. Difference was considered significant when the *p* value is less than 0.05.

## Results

### Brain Proinflammatory Responses during CLP-induced Sepsis

To estimate proinflammatory cytokine levels associated with brain inflammation during CLP-induced sepsis, we initially performed real-time PCR analysis from total RNA isolated from brain and lung tissues of sham-operated control and CLP mice. Sepsis induced by CLP resulted in a significant up-regulation of cerebral expression of TNF-α and IL-1β mRNA (*n* = 5) (Fig. 1A). The results of quantitative analysis showed up to 3.6-fold and 2.6-fold respectively, in brain tissues at 24 h after CLP. IL-6 mRNA level in brain tissues was also increased 1.6-fold at 12 h after CLP, although no statistical difference was found. Induction of sepsis by CLP also led to time-dependent increases in protein expression of these proinflammatory cytokines (Fig. 1B). It should be noted, however, that the extent of the increase in mRNA expression of these pro-inflammatory cytokines in brain tissues was far less than that observed in lung tissues (*n* = 3) (Fig. 1C).

The BBB, formed primarily by cerebrovascular endothelial cells, strictly restricts the transport of ions, amino acids, peptides, and other substances from the peripheral circulation to the CNS. The BBB integrity may be disrupted during the course of various inflammatory disorders in the CNS. We assessed sepsis-induced changes in the permeability of the BBB to small and large molecules quantitatively using intravenous sodium fluorescein and Evans blue, respectively. At 24 h after the onset of CLP-induced sepsis, the sodium fluorescein content within the brain was significantly increased when compared with sham-operated controls (*n* = 4) (Fig. 2A). Similarly, the extravasation of Evans blue into brains of septic mice was significantly greater than that in controls (*n* = 6) (Fig. 2B).

### Brain Histopathologic Changes after CLP-induced Sepsis

Histopathologic analysis of the sections stained with hematoxylin and eosin revealed that the morphology of neurons in the cerebral cortex was normal in the sham group (Fig. 3A). In contrast, serious neuronal degeneration was observed in cerebral cortex sections from mice at 24 h after CLP (Fig. 3B). Numerous hyperchromatic cells were found. The nuclei were darker than the perikarya. The cytoplasm was shrunken with perineuronal vacuolations. The hippocampus appeared to be the most affected area (Fig. 4).

Fig. 5 displays electron micrographs of cerebral cortices from sham-operated control and CLP septic mice. Electron microscopic analysis indicated the intact structure of the brain capillary in the sham-operated control group (Fig. 5A). Neighboring neuronal and glial processes appeared normal. On the other hand, brain capillary endothelial cells were swollen and their cell membranes were ruptured in the CLP septic group (Fig. 5B). Furthermore, there was found a neuron with increased electron density of its cytoplasm and karyoplasm (Fig. 5C). In the nucleus, dispersed chromatin was observed. Its cytoplasm contained numerous irregularly dispersed vacuoles with degenerated organelles.

### Changes in Oxidative Stress Markers after CLP-induced Sepsis

Fig. 6A shows the time course of iNOS protein expression in brains after CLP. Expression of iNOS protein was detected at a low level in control brains. Sepsis induction by CLP up-regulated cerebral expression of iNOS protein in a time-dependent manner. At 24 h after CLP, iNOS protein was increased 1.8-fold compared with sham-operated controls (*n* = 3) (Fig. 6B and C).

NADPH oxidase activity was time-dependently elevated in brains after CLP (*n* = 6) (Fig. 7A). The brain tissues from septic mice at 20 h after CLP had a 2.3-fold greater level of NADPH oxidase activity than controls. Higher NADPH oxidase activity was also reflected at the expression levels of NADPH oxidase subunits. Thus, Western immunoblotting showed that NADPH oxidase components p47*^phox^* and p67*^phox^* were up-regulated in brains from CLP-induced septic mice (Fig. 7B). Quantitative analysis of p47*^phox^* and p67*^phox^* mRNA expression by real-time PCR confirmed a significant increase in cerebral expression of p47*^phox^* and p67*^phox^* in septic mice at 6 h after surgery compared with sham-operated controls (*n* = 6) (Fig. 7C).

Determination of 8-OHdG (Fig. 7D), an indicator of oxidative DNA damage, showed that it was significantly increased in brains from CLP-induced septic mice compared with sham-operated controls.

### Preventive Effect of Edaravone on Septic Histopathologic Changes of Brains

The cerebral levels of 8-OHdG in 24-h septic mice were significantly (*p*<0.05) reduced from 18.4±1.5 (*n* = 7) to 11.8±2.4 ng/mg DNA (*n* = 7) by edaravone treatment. Treatment with edaravone also resulted in a significant decrease in the sodium fluorescein content within the brain at 24 h after sepsis (111±19 vs. 56±9 ng/ml, *n* = 6, *p*<0.05).

The histological evaluation of brain tissues with hematoxylin and eosin did not show any morphological difference between the sham-operated control animals treated with edaravone and its vehicle. Treatment with edaravone resulted in a preventive effect on the degenerative neuronal changes in cerebral cortex and hippocampal areas observed in mice at 24 h after CLP-induced sepsis (Figs. 8 and 9).

## Discussion

The release of endotoxin from bacteria is generally believed to be the initial event in the development of sepsis. Endotoxin activates inflammatory cells of the myeloid lineage that subsequently amplify the inflammatory response by releasing proinflammatory cytokines. In this study, we demonstrated a significant increase in gene production of proinflammatory cytokines, including TNF-α and IL-1β, in brain tissues of mice at 12–24 h after sepsis induction by CLP. This suggests that septic inflammatory response spreads to the brain in our CLP mouse model, although the up-regulated levels of proinflammatory cytokine genes in brains were far from those in peripheral tissues such as lungs.

The BBB is highly restrictive of the transport of substances between blood and the CNS. The BBB is primarily formed by cerebrovascular endothelial cells that are sealed with tight junctions [25]. Disruption of the BBB integrity can occur as a consequence of sepsis [8], [26]. For quite a while, BBB dysfunction has been described in a rodent model of sepsis and in patients with septic encephalopathy [27]–[30]. Moreover, it has been shown that high protein levels in cerebrospinal fluid and perimicrovessel edema are notable features of cerebral damage in patients with septic encephalopathy [31], [32], suggesting that integrity of BBB during human sepsis is impaired. In the current study, we measured BBB permeability in CLP-induced septic mice using two cellular transmarkers, sodium fluorescein and Evans blue. Extravasation of these dyes provided quantitative information about vascular permeability to relatively small and large molecules. Permeability of sodium fluorescein and Evans blue confirmed the leakiness of the BBB 24 h after sepsis induction. The difference in the permeability of the BBB for the two dyes with different molecules was not observed. In addition, swelling of brain capillary endothelial cells and rupture of their cell membranes in septic mice were clearly demonstrated by our present electron microscopic analysis. Consequently, opening of BBB could lead to vasogenic brain edema and subsequently brain damage [32], [33].

Our histopathologic analysis showed serious neuronal degeneration in septic mouse brains. Hyperchromatic, shrunken, and pyknotic neurons were found. Ultrastructurally, increased electron density was regarded as a darkened nuclear and cytoplasmic matrix, and the cytoplasmic organelles exhibited highly degenerative changes. Histopathological changes were widespread but the hippocampus appeared to be the most affected region. The hippocampus injury could explain recognition memory impairment in patients with septic encephalopathy [34], since this area is critically involved in memory forming, organizing, and storing. To the best of our knowledge, this is the first work that reported rapid neuronal degeneration in brains after induction of sepsis by CLP morphologically.

One of the key features of sepsis is an increased production of reactive oxygen species (ROS) in the affected organs, including brain [35]–[37]. ROS is known for its deleterious effects, which can cause enhanced permeability of the BBB [38], [39]. It has been documented that cerebrovascular endothelial cells are enriched in NADPH oxidase and, therefore, can produce high quantities of ROS [40], thus potentially contributing to the impaired function of the BBB. The present study demonstrated that both NADPH activity and NADPH oxidase components p47*^phox^* and p67*^phox^* were enhanced in septic mouse brains. We also showed that cerebral expression of iNOS protein was significantly up-regulated in septic animals. Within the CNS, microglia and astrocytes can generate nitric oxide (NO) radicals from iNOS activation [41], [42]. NO produced in large quantities by iNOS is thought to be a damaging radical, which might be responsible for the oxidative/nitrosative brain injury [43]. Interestingly, our immunofluorescent study identified protein tyrosine nitration induced by peroxynitrite, a reaction product of superoxide anions with NO, each of which is generated by NADPH oxidase and iNOS, respectively, in cerebral vessels of septic mice (unpublished observations). Nitrated tyrosine has been detected at lesion sites following BBB breakdown in rat brain trauma and in postmortem tissues of multiple sclerosis patients [44]. Evidence for nitration of tyrosine as an indicator of peroxynitrite has also been provided in other brain inflammatory disorders in which there are disturbances at the BBB [45], [46]. Furthermore, uric acid, a natural peroxynitrite scavenger molecule, can prevent pathological CNS inflammation through maintenance of blood-CNS barrier integrity [47], [48]. Taken together, these reports implicate peroxynitrite in the loss of BBB integrity in many neuroinflammatory disorders. Hence, we propose that, during sepsis, accelerated peroxynitrite generation may occur in brains to mediate cerebrovascular endothelial cell injury and thus breakdown of BBB integrity, which could possibly lead to widespread neuronal degeneration.

The principal finding of this work is that neuronal degenerative changes seen in septic mouse brains were nearly completely normalized when the animals were treated with edaravone. Edaravone, a newly developed free radical scavengers for clinical use, has a potent free-radical-quenching action by trapping a variety of free radical species [49], and appears to be very effective in preventing cerebral damage in patients with cerebral infarction [50]. Furthermore, edaravone has been shown to alleviate delayed neuronal death after cerebral ischemia-reperfusion injury in rats [51]. It seems reasonable, therefore, to assume that edaravone could prevent sepsis-associated neuronal degeneration by scavenging free radicals generated in diseased brain tissues. In light of the possible role of peroxynitrite as referred to above, the beneficial effect of edaravone treatment may be primarily a result of prevention of peroxynitrite generation leading to protect from BBB disruption. However, additional work is needed to delineate the precise molecular mechanism(s) responsible for the benefit of edaravone to provide neuroprotection from sepsis pathology.

In conclusion, we demonstrated that CLP-induced septic mice displayed morphologically rapid and widespread neuronal degeneration in brains. The present results indicate that oxidative and nitrosative stresses may prove the molecular basis underlying CNS dysfunctions, including the disruption of the BBB, in sepsis. Treatment with edaravone prevented histopathologic outcomes after sepsis in mice. This supports the significance of free radical species in sepsis-associated CNS alternations and provides a potential new approach to the treatment of septic encephalopathy.
